# Supplementary material for: The potential mechanisms of reciprocal regulation of gut microbiota-liver immune signaling in metabolic dysfunction-associated steatohepatitis revealed in multi-omics analysis
Source: mSystems. 2025 Jun 10;10(7):e00518-25. doi: 10.1128/msystems.00518-25 (PMC12282060; doi:10.1128/msystems.00518-25)

**Supplementary materials**

**Table S1**

| **Immune-and inflammation-related GO-BP in GSEA of DEG (NES>1.8)** | | | | | |
| --- | --- | --- | --- | --- | --- |
| Description | setSize | enrichmentScore | NES | pvalue | p.adjust |
| adaptive immune response | 512 | 0.503 | 2.07 | 8.56e-20 | 5.45e-16 |
| immune effector process | 701 | 0.487 | 2.04 | 6.57e-24 | 4.18e-20 |
| chronic inflammatory response | 19 | 0.773 | 1.98 | 5.64e-05 | 0.3593 |
| antifungal innate immune response | 18 | 0.777 | 1.973 | 0.00019 | 1 |
| regulation of immune response | 869 | 0.460 | 1.96 | 3.06e-23 | 1.95e-19 |
| positive regulation of adaptive immune response | 143 | 0.535 | 1.95 | 5.84e-08 | 0.000372 |
| positive regulation of adaptive immune response based on somatic recombination of immune receptors built from immunoglobulin superfamily domains | 135 | 0.538 | 1.95 | 8.21e-08 | 0.0005 |
| adaptive immune response based on somatic recombination of immune receptors built from immunoglobulin superfamily domains | 396 | 0.485 | 1.95 | 1.07e-13 | 6.79e-10 |
| regulation of immune effector process | 410 | 0.479 | 1.93 | 5.84e-14 | 3.72e-10 |
| immunoglobulin mediated immune response | 225 | 0.500 | 1.93 | 1.58e-09 | 1.01e-05 |
| production of molecular mediator of immune response | 344 | 0.482 | 1.93 | 1.90e-11 | 1.21e-07 |
| negative regulation of immune response | 210 | 0.506 | 1.93 | 2.92e-09 | 1.86e-05 |
| positive regulation of immune effector process | 265 | 0.492 | 1.93 | 8.38e-10 | 5.34e-06 |
| innate immune response | 838 | 0.452 | 1.92 | 1.16e-20 | 7.42e-17 |
| production of molecular mediator involved in inflammatory response | 94 | 0.562 | 1.92 | 1.44e-06 | 0.009201 |
| positive regulation of inflammatory response | 144 | 0.526 | 1.92 | 2.28e-07 | 0.001455 |
| cytokine production involved in immune response | 152 | 0.518 | 1.91 | 3.42e-07 | 0.002179 |
| regulation of adaptive immune response | 217 | 0.496 | 1.90 | 9.74e-09 | 6.21e-05 |
| regulation of innate immune response | 442 | 0.464 | 1.90 | 5.69e-13 | 3.63e-09 |
| inflammatory response | 760 | 0.443 | 1.88 | 1.96e-17 | 1.25e-13 |
| regulation of inflammatory response | 355 | 0.467 | 1.87 | 1.31e-10 | 8.35e-07 |
| leukocyte activation involved in immune response | 281 | 0.475 | 1.86 | 3.98e-09 | 2.54e-05 |
| cell activation involved in immune response | 285 | 0.473 | 1.85 | 3.92e-09 | 2.50e-05 |
| regulation of adaptive immune response based on somatic recombination of immune receptors built from immunoglobulin superfamily domains | 201 | 0.488 | 1.85 | 1.02e-07 | 0.000648 |
| regulation of cytokine production involved in immune response | 133 | 0.513 | 1.85 | 1.59e-06 | 0.010133 |
| positive regulation of immune response | 706 | 0.437 | 1.84 | 7.01e-16 | 4.47e-12 |
| myeloid cell activation involved in immune response | 96 | 0.529 | 1.82 | 3.85e-05 | 0.245667 |
| inflammatory response to antigenic stimulus | 77 | 0.552 | 1.82 | 6.77e-05 | 0.431205 |

**Table S2.**

| **18 pathways common to the transcriptome and metabolome** | | | | |
| --- | --- | --- | --- | --- |
| ID | Desciption | pvalue | p.adjust | Count |
| map00591 | Linoleic acid metabolism | 7.536e-05 | 0.01017 | 7 |
| map00260 | Glycine, serine and threonine metabolism | 0.001018 | 0.03434 | 5 |
| map00564 | Glycerophospholipid metabolism | 0.002051 | 0.05537 | 12 |
| map00120 | Primary bile acid biosynthesis | 0.006949 | 0.1173 | 4 |
| map00380 | Tryptophan metabolism | 0.01108 | 0.136 | 5 |
| map01232 | Nucleotide metabolism | 0.01445 | 0.1625 | 4 |
| map00330 | Arginine and proline metabolism | 0.02574 | 0.2317 | 4 |
| map00640 | Propanoate metabolism | 0.02882 | 0.2289 | 3 |
| map00592 | alpha-Linolenic acid metabolism | 0.03458 | 0.2457 | 5 |
| map00220 | Arginine biosynthesis | 0.05365 | 0.3018 | 2 |
| map00051 | Fructose and mannose metabolism | 0.06307 | 0.3275 | 3 |
| map00630 | Glyoxylate and dicarboxylate metabolism | 0.08653 | 0.2717 | 3 |
| map00240 | Pyrimidine metabolism | 0.08653 | 0.2717 | 3 |
| map00410 | beta-Alanine metabolism | 0.09582 | 0.2488 | 2 |
| map01040 | Biosynthesis of unsaturated fatty acids | 0.1202 | 0.2751 | 3 |
| map00480 | Glutathione metabolism | 0.1277 | 0.2737 | 2 |
| map00590 | Arachidonic acid metabolism | 0.1387 | 0.2637 | 5 |
| map00270 | Cysteine and methionine metabolism | 0.3005 | 0.3757 | 2 |

**Figure S1. Stacked histograms of relative abundance of gut microbiota at the species level**


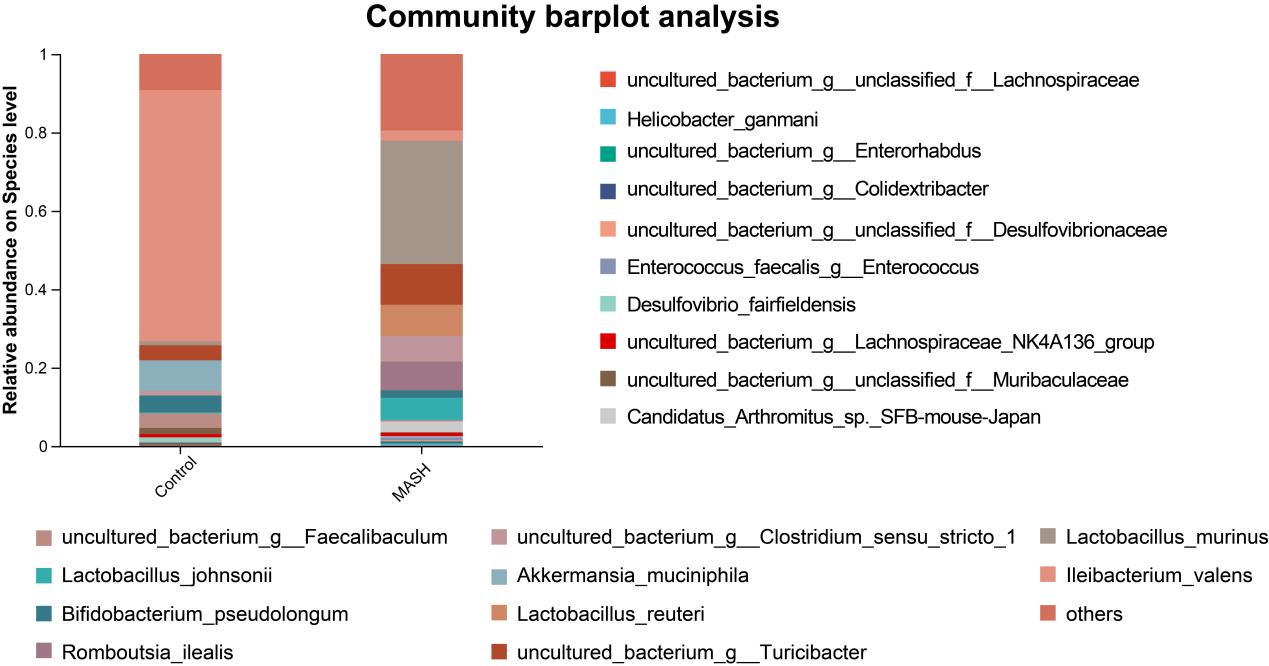


**Figure S2. Intergroup correlation networks were generated for all variables in the liver transcriptome, serum metabolome, and genus-level gut flora for both groups**


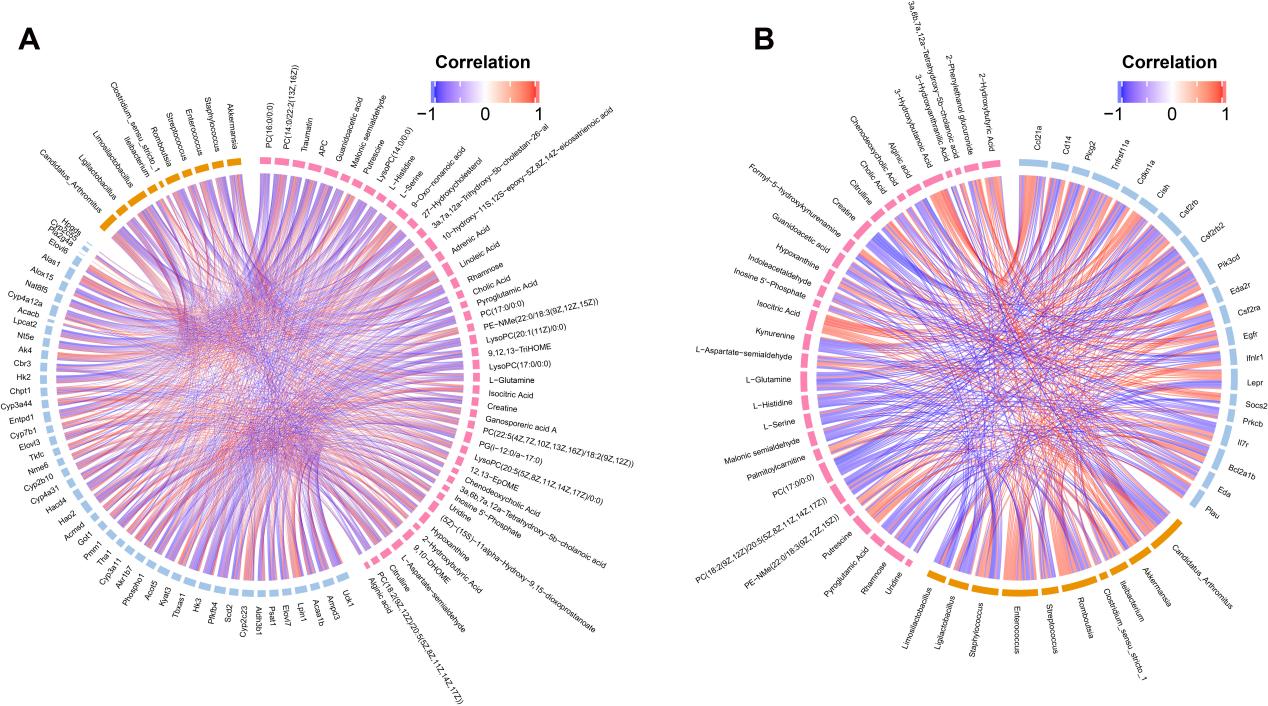


A. Intergroup correlation networks were generated for all variables of the liver transcriptome, serum metabolome, and genus-level gut microbiota that may suggest potential regulation of metabolism. B. Intergroup correlation networks were generated for all variables of the liver transcriptome, serum metabolome, and genus-level gut microbiota that may suggest potential regulation of immunity. The red line indicates a positive correlation (r ≥ 0.81); The blue line indicates a negative correlation (r ≤ - 0.81), and the size of nodes was proportional to the degree of connectivity with other nodes in the network.

**Figure S3. Differential analysis of the results of gene function annotation of the gut microbiota.**


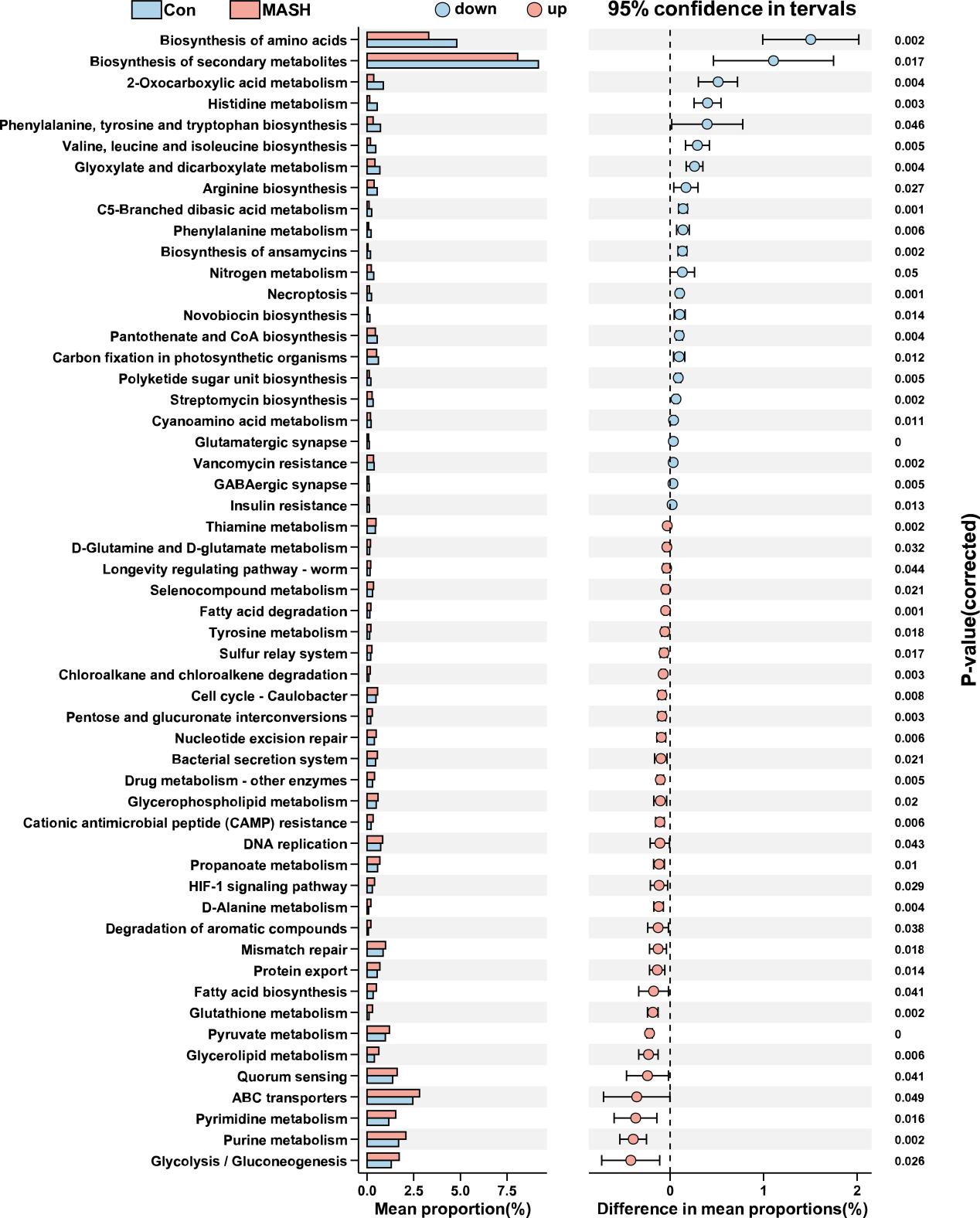

Supplement: Supplemental material — Tables S1 and S2; Fig. S1 to S3. [file msystems.00518-25-s0001.docx]
