# Supplementary material for: The potential mechanisms of reciprocal regulation of gut microbiota-liver immune signaling in metabolic dysfunction-associated steatohepatitis revealed in multi-omics analysis
Source: mSystems. 2025 Jun 10;10(7):e00518-25. doi: 10.1128/msystems.00518-25 (PMC12282060; doi:10.1128/msystems.00518-25)
Supplement: Approval — IACUC approval document. [file msystems.00518-25-s0002.pdf]

哈尔滨医科大学伦理审查报告
（科研项目申请专用）

项目名称	唾液链球菌通过PPARG介导LIFR调控肝硬化进展的机制研究		
项目负责人	金也	职称	助理研究员
电子邮箱	40897106@qq.com	电话	13936398339
所在单位	哈尔滨医科大学附属第一医院		
研究材料	☑永生细胞系 ☑动物 ☑医学信息 ☑人类废弃标本 ☑人或人群		
材料来源	☑购买 ☐公开获取 ☐去标识后获取 ☑临床采集 ☑受试者招募		
提交资料	☑研究方案 ☑知情同意书（样本）		

审 查 结 论

根据《世界医学协会赫尔辛基宣言》、《涉及人的生物医学研究伦理审查办法》等国内外相关伦理规范，哈尔滨医科大学附属第一医院伦理委员会对本项目进行了伦理审查。本次审查伦理委员会适用简易程序，在且仅在对项目申请人提交的研究方案、知情同意书进行审查后认为：该项目在且仅在项目设计、申报中，对研究涉及的受试者保护、风险收益比评估等伦理问题给予了足够关切并进行了合理的设计，符合伦理要求，同意申报本项目。

经上级主管部门同意立项后，进行伦理会审，取得伦理委员会批件后，遵照提交给伦理委员会的研究方案开展相关工作。

伦理委员会意见	主审专家	副审专家	伦理/法律/社会专家
	[illegible]	[illegible]	[illegible]
	伦理委员会主任签字：[illegible]	伦理委员会公章：哈尔滨医科大学附属第一医院 伦理委员会	2024 年 2 月 21 日

本报告一式两份，正本用于伦理委员会存档，副本用于项目申报。
